# Ruptured pulmonary hydatid cyst: a rare clinical image

**DOI:** 10.11604/pamj.2024.47.136.42754

**Published:** 2024-03-25

**Authors:** Ashwin Karnan

**Affiliations:** 1 Department of Respiratory Medicine, Jawaharlal Nehru Medical College, Datta Meghe Institute of Higher Education and Research, Sawangi (Meghe), Wardha, Maharashtra, India

**Keywords:** Cyst, cough, hydatid, albendazole

## Image in medicine

A 43-year-old female presented to the respiratory medicine outpatient department with complaints of dry cough, fever, and breathlessness for the past 3 months. The patient has a salty taste of saliva with no comorbidities and no significant past or personal history. A computed tomography scan of the thorax showed well-defined multiple rounded cystic lesions on the right lower lobe with internal air-fluid level with moderate loculated hydropneumothorax. Serum IgG for echinococcus granulosus was positive. The patient was started on Tablet Albendazole 400mg twice daily. Echinococcosis is a rare infectious disease that occurs mainly due to echinococcus granulosus or echinococcus multilocularis. The liver is the most common site of infection followed by the lungs, spleen, kidneys, heart, and bone. Cysts may rupture leading to secondary infection, suppuration, or pneumothorax. Immuno electrophoresis, ELISA, and hemagglutination are serological evidence of echinococcosis. They may be treated surgically or pharmacologically. Albendazole is the drug of choice given for at least 3-6 months. Enucleation, pericystectomy, cystotomy, and segmental resection are the surgical methods available.

**Figure 1:**
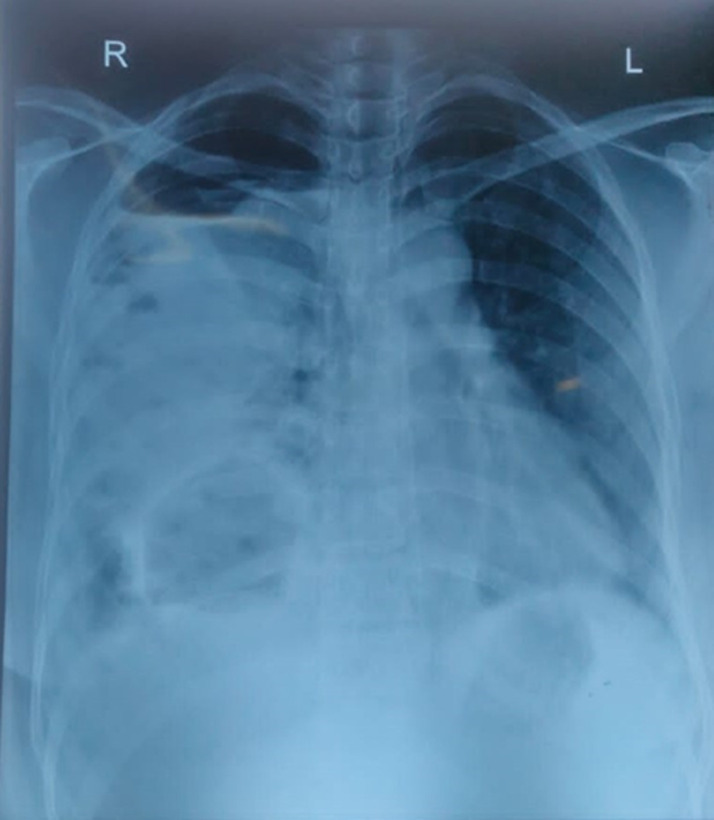
chest X-ray showing cyst in right lung lower lobe with right hydro pneumothorax

